# Antibacterial and Antibiofilm Activities of* Cinnamomum* Sp. Essential Oil and Cinnamaldehyde: Antimicrobial Activities

**DOI:** 10.1155/2018/7405736

**Published:** 2018-06-06

**Authors:** Diego F. Firmino, Theodora T. A. Cavalcante, Geovany A. Gomes, Nairley C. S. Firmino, Lucas D. Rosa, Mário G. de Carvalho, Francisco E. A. Catunda Jr

**Affiliations:** ^1^University Center UNINTA, Exact Sciences Center, Sobral, CE, Brazil; ^2^University Center UNINTA, Medical Sciences Center, Sobral, CE, Brazil; ^3^Acarau Valley State University, Center for Science and Technology, Sobral, CE, Brazil; ^4^University Center UNINTA, Health Sciences Center, Sobral, CE, Brazil; ^5^Federal Rural University of Rio de Janeiro, Exact Sciences Center, Seropédica, RJ, Brazil; ^6^State University of the Tocantina Region of Maranhão, Center for Exact, Natural and Technological Sciences, Imperatriz, MA, Brazil

## Abstract

To assess the activities of essential oils derived from the trunk bark of* Cinnamomum zeylanicum* (EOCz) and* Cinnamomum cassia* (EOCc) as well as cinnamaldehyde on bacterial biofilms of clinical interest. Antimicrobial activity was assessed by the broth microdilution method to determine minimum inhibitory concentrations (MICs). Antibiofilm activity was assessed by quantifying the biomass and determining the number of viable cells. The chemical composition of the essential oils was determined. The results showed that the major component of EOCz and EOCc was cinnamaldehyde. For the assayed substances, biofilm biomasses were reduced by up to 99.9%, and* Streptococcus pyogenes*,* Pseudomonas aeruginosa*, and* Escherichia coli* biofilms were sensitive to all of the concentrations and substances analysed. In cell viability tests, 2 mg/ml of cinnamaldehyde reduced the number of viable cells by 5.74 Log CFU/ml. EOCz, EOCc, and cinnamaldehyde exhibited antimicrobial and antibiofilm activities. This work describes substances with potential use against infections caused by bacterial biofilms.

## 1. Introduction

Biofilms are organized groups of microorganisms that live inside an extracellular polymer matrix that is self-sustaining and can adhere to various surfaces [1], biotic and abiotic [2]. Biofilms are often observed floating or submerged in liquids [3] and can be composed of homogeneous or heterogeneous communities of bacteria within a polymer matrix, which is primarily composed OF polysaccharides as well as other biomolecules, such as proteins, lipids, and nucleic acids [2]. Analyses of the polymer matrix have revealed that biofilms are hydrogels that exhibit viscoelastic behavior, allowing them to resist mechanical stresses [4].

Many biofilms are present in a variety of microbial infections, including dental infections [5]; periodontitis [6]; lung infections resulting from cystic fibrosis and facial filling [7]; chronic wounds [8]; ear inflammation [9]; implant-associate infections [10]; chronic rhinosinusitis [11]; contamination in intensive care units (ICU) [12]; contact lens infections [13]; and human gastrointestinal tract infections [14]. Studies have shown that* S. aureus* is the second most prevalent pathogen in ICUs [15] and often causes infections in women, with biofilms formed by this microorganism causing complications in urinary infection treatments [16].

Biofilms protect microorganisms from external aggression and predator attacks [17], and some populations of biofilm-associated bacteria are resistant to antibiotics [5]. Thus, the increased resistance of bacterial biofilms to antibiotics is problematic for the use of antimicrobial drugs [18], driving the search for alternative medicinal plants that can be used to treat diseases [19]. In particular, it is necessary to identify new drugs that can serve as an alternative treatment of infections caused by microorganisms that are resistant to traditional therapies. One approach is the study of local medicinal plants with possible antimicrobial and antibiofilm properties.

Essential oils, which are natural products obtained from plants, contain volatile organic compounds that can be obtained from various parts of the plant, such as flowers, fruits, seeds, stems, AND roots [20]. These oils have demonstrated antioxidant, insecticidal, antiviral, antibacterial, antifungal, and antibiofilm activities [21–23]. Among essential oils of botanical origin that have antimicrobial potential are those obtained from plant species of the genus* Cinnamomum* (Lauraceae), such as* C. zeylanicum *and* C. cassia.*

*C. zeylanicum *is native to some regions of India and Ceylon; thus, it is known as “cinnamon-of-ceylon” [24]. This species has been used in folk medicine due to its many medicinal properties, including its activities as astringent, aphrodisiac, antiseptic, aperitif, aromatic, carminative, digestive, stimulant, hypertensive, sedative, tonic and vasodilator [25], antidiabetic, antinociceptive, astringent, and diuretic [26]. From the different parts of the plant, essential oils with various chemical compositions can be obtained, and essential oil from the stem bark contains up to 4% oil consisting of (E)-cinnamaldehyde (65-78%) and eugenol (4-10%), accompanied by low percentages of cinnamyl acetate, methyl-n-amyl-ketone, and mono- and sesquiterpenoids [27].

*C. cassia, *popularly known as “cinnamon-china”, is a perennial tree native to southern China and is widely cultivated in southern and eastern Asia (Taiwan, Laos, Thailand, Vietnam, India, Indonesia, and Malaysia) [28].* C. cassia *is used in Western medicine, primarily in the treatment of diarrhoea, flatulent dyspepsia, cramps, colds, flu, cough, bronchitis, lack of appetite, renal weakness, arthritic angina, palpitations, spasms, vomiting, gastric ulcers, digestive complaints, and bacterial and fungal infections of the skin [29]. The trunk bark contains at least 1.0% essential oil, consisting of 70 to 90% (E)-cinnamaldehyde [30]. In addition, a study also reported that the essential oil of this species has antifungal activity against* Candida albicans *[31].

(E)-Cinnamaldehyde, the most abundant component of the essential oils of the* Cinnamomum *species mentioned, is a phenylpropanoid that has proven activity against microorganisms [32]. Due to this already proven antimicrobial action, this work aimed to verify the action of essential oils and cinnamaldehyde against microbial biofilms of clinical interest.

## 2. Materials and Methods

### 2.1. Essential Oil

Two 10 ml units of essential oils, extracted from* C. cassia *and* C. zeylanicum* trunk bark by steam distillation, were purchased from Lazlo® (Belo Horizonte, MG), and (E)-cinnamaldehyde was purchased from Sigma-Aldrich®.

### 2.2. Chemical Analysis of Essential Oils

The chemical compositions of the essential oils were analysed by gas chromatography coupled to a mass spectrometer (GC-MS) using a Shimadzu QP2010 Plus chromatograph with helium (He) as the carrier gas and a FactorFour/VF-5 ms capillary column (30 m long, 0.25 mm internal diameter, and 0.25 mm film thickness). The carrier gas was used at a flow rate of 1 ml/min. The initial oven temperature was 60°C, followed by constant heating for 2 min, and was increased 2°C per minute to 110°C, 3°C per minute to 150°C, and 15°C per minute to 290°C, with a final isotherm of 290°C for 17 minutes. The temperatures of the injector and detector were, respectively, set at 250°C and 310°C. The injection mode was split and the injection volume used was 1 *μ*l. The mass spectra were produced by electron impact (70 eV).

Quantitative analyses of the chemical compositions of the essential oils were performed on a gas chromatograph coupled to an HP5890 Series II ionization detector (GC-FID), using the same operating conditions and the same column type as was used in the GC/MS analysis, except the temperatures of the injector and detector which were set at 220°C and 250°C, respectively.

The percentage of each constituent was calculated by the integral of the area of the respective peaks in relation to the total area of all constituents of the sample. The various constituents of the essential oil were identified by visual comparison of their mass spectra with those previously published and with standards from the Nist08 library on the instrument, as well as by comparing retention indices with those previously published. A standard solution of n-alkanes (C8-C20) was injected under the same chromatographic conditions of the sample and used to obtain the retention indices [33].

### 2.3. Substances and Preparation of Solutions

The substances used in the tests were included the essential oils from the trunk bark of* C. cassia* and* C. zeylanicum*, cinnamaldehyde, and oxacillin as a positive control. Calculations for the preparation of the solutions containing the oils and cinnamaldehyde were performed on the basis of the density of the substances and, to facilitate solubilization, 2% Tween 20 was used. All solutions were prepared at an initial concentration of 4 mg/ml and, after being diluted in broth, the resulting concentrations ranged from 2 to 0.01 mg/ml for all substances.

### 2.4. Bacterial Strains and Culture Conditions

The bacteria used in this study were included* S. aureus* ATCC6538,* S. epidermidis* ATCC12228,* S. pyogenes* ATCC19615,* P. aeruginosa *ATCC15442, and* E. coli* ATCC11303. The strains were maintained in BHI (Brain Heart Infusion-Difco®) + glycerol (20%) at -80°C. To perform the experiments, a 100 *μ*l aliquot was inoculated onto TSA medium (*Trypticase Soy Agar*-Difco®) and grown in a greenhouse at 37°C for 24 h. After this initial growth, a 100 *μ*l inoculum was subcultured into 10 ml of TSB (*Trypticase Soy Broth*- Difco®) and grown for 18 h under the same conditions described above. In the antimicrobial activity assays, the cultures were washed with Mili-Rios water and their concentrations were adjusted to 10^7^-10^8^ CFU/ml using a microplate reader (SpectraMax i3 Multi-Mode Microplate Reader®) at 620 nm.

### 2.5. Antimicrobial Activity

#### 2.5.1. Minimum Inhibitory Concentration

The antimicrobial activity of the* Cinnamomum* stem essential oils against the five bacterial strains in their planktonic forms was tested by the microdilution method in polystyrene plates. The minimum inhibitory concentration was determined by the microdilution technique in 96-well polystyrene plates. For the assembly of the plates, 100 *μ*l of the essential oil solutions was serially diluted in culture medium, with the final concentrations varying from 2.00 to 0.01 mg/ml. Next, 100 *μ*l of culture medium containing the microorganisms was added at the adjusted concentration of 10^7^-10^8^ CFU/ml as described above. The negative control consisted of growing the microorganisms in TSB culture medium with 2% Tween 20. The MIC was considered the lowest concentration of the substances at which no growth of the microorganisms was detected [34].

#### 2.5.2. Minimum Bactericidal Concentration

The minimum bactericidal concentration was determined by withdrawing a 10 *μ*l aliquot of the bacterial suspensions at concentrations where no visual growth of the microorganisms was observed. The aliquot was inoculated into Petri dishes containing TSA and incubated in an oven at 37°C, in triplicate, with bacterial growth observed after 24 h [34].

### 2.6. Antibiofilm Activity

#### 2.6.1. Quantification of Biofilm Biomass

The plates were assembled in a process similar to the MIC test, and after 24 h the antibiofilm activity was assessed through the methods mentioned and described below. After 24 h incubation, the plates were washed with sterile water (200 *μ*l/well) to remove the loosely adhered and air-dried cells. The adhered cells of the biofilm were fixed in the plate wells by addition of 200 *μ*l of methanol for 15 min. Afterwards, the methanol was removed and 200 *μ*l of a 1% violet crystal solution was added for 15 min. Subsequently, the plates were washed and air dried again, after which 200 *μ*l of ethanol (96%) was added to each well which were left shaking for 5 min and then read on a microplate reader at 595 nm [35].

#### 2.6.2. Verification of Cell Viability in the Biofilm

After the incubation period, the culture medium was removed and the plates were subjected to three washes with distilled water. Next, 200 *μ*l of a 0.9% saline solution was aliquoted into the wells, and the plate was incubated in a sonic bath (LK-D32 Ultrasonic Bath) operating at 50 kHz for 10 min. The liquid from the wells for each concentration teste was pooled to bring the volume to 1 ml, after which a 20 *μ*l aliquot was withdrawn and subjected to serial dilution in 180 *μ*l volumes of 0.85% saline solution (10^−1^ to 10^−7^). In a Petri dish containing TSA, three 10 *μ*l aliquots were cultured for each concentration. These plates were incubated for 24 h at 37°C, after which the resulting colonies were counted [35].

### 2.7. Statistical Analysis

Statistical evaluation of the data was performed with the program GraphPad®, San Diego California, USA, version 5.0. The statistical test used was ANOVA for multiple comparisons, followed by the Bonferroni test. Values of p <0.01 were considered statistically significant and are indicated by an asterisk. Each MIC experiment and indirect biofilm biomass quantification was performed with five replicates, and the CBM tests and CFU counts were performed using three replicates. All tests were performed in three independent experiments.

## 3. Results and Discussion

### 3.1. Chemical Composition of Essential Oils

**Figure 1:**
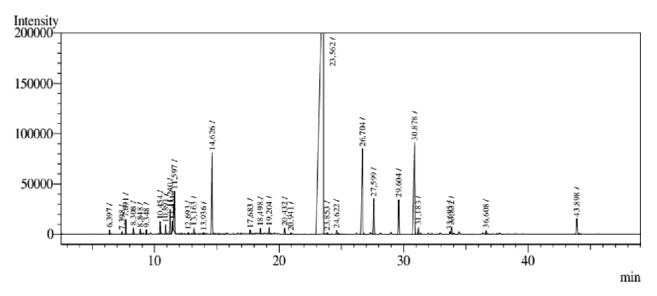
GC-MS chromatogram analysis* C. zeylanicum* essential oil stems (EOCz).

**Figure 2:**
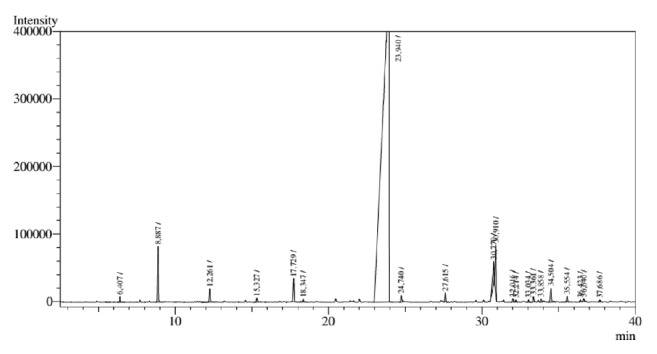
GC-MS chromatogram analysis* C. cassia* essential oil stems (EOCc).

The analysis of the chemical composition of the essential oils showed their chemical constituents and the percentage composition of the essential oils. According to the data presented in Table 1, the EOCz contained 31 compounds, among which (E)-cinnamaldehyde, cinnamoyl (E)-acetate, and eugenol were found in the highest percentages (68.7, 71.2, and 6.33%, respectively). In the EOCc, 18 substances were detected, with the major component being (E)-cinnamaldehyde, corresponding to 90.22% of the total components. The chromatograms are in Figures 1 and 2.

A study carried out in Iran using* C. zeylanicum* stalks extracted by hydrodistillation revealed the presence of 17 compounds in the extracted essential oil. The major groups of compounds were monoterpene hydrocarbons and phenolic compounds. Cinnamaldehyde (80.42%), *α*-copaene (2.73%) and trans-calamenene (2.16%) were the primary chemical constituents of the oil. Other components analysed in the oil were present at amounts below 2% [36].

In a study to evaluate the chemical composition of the essential oil of* C. cassia* bark obtained from the lower part of the stems at eight growth stages, most of the identified compounds observed belonged to the sesquiterpene hydrocarbon and oxygenated sesquiterpene fractions at all stages. The major component identified was (E)-cinnamaldehyde [37].

The factors that determine the yield and composition of the essential oils are numerous. In some cases, it is difficult to isolate these factors from each other as they are interrelated and influence each other. These parameters include seasonal variations, the organ of the plant used, and the maturity of the plant, as well as its geographic origin and genetics [38].

### 3.2. Antimicrobial Activity of* C. zeylanicum* and* C. cassia* Bark Essential Oils and (E)-Cinnamaldehyde on Bacteria

Essential oils and (E)-cinnamaldehyde inhibited the growth of all bacteria assayed in this study in planktonic form, the antimicrobial activity data for which are shown in Table 2.

The EOCz, EOCc, and (E)-cinnamaldehyde exhibited bacteriostatic and bactericidal activity against* S. aureus* and* S. epidermidis*. The MIC values ranged from 0.25 to 0.50 mg/ml, and the EOCz showing a bacteriostatic effect at 0.50 mg/ml, while EOCc and (E)-cinnamaldehyde had the same effect at 0.25 mg/ml. At the concentrations assayed in this study, no MIC or MBC for oxacillin could be determined, since growth was not detected at any concentration tested. The similarity between the data can be explained by the amount of (E)-cinnamaldehyde present in the EOCc (90.22%) (see Table 1). The MIC value for the oils and (E)-cinnamaldehyde against* S. pyogenes* and* P. aeruginosa* was 0.50 mg/ml, and no bactericidal effect of these natural products was observed for these bacteria. In contrast, for* E. coli*, only EOCc did not exhibit a bactericidal effect, and the MIC varied from 0.25 to 0.50 mg/ml, and the MBC was 2.00 mg/ml for both for EOCZ and (E)-cinnamaldehyde.

In a study by [39], it was shown that the essential oil of* C. cassia* was 85.06% (E)-cinnamaldehyde. In addition, antimicrobial activity was found against strains of* P. aeruginosa, E. coli,* and* S. aureus*. The results demonstrated a strong inhibitory effect in which the MICs of (E)-cinnamaldehyde and* C. cassia *oil were 0.3 mg/ml for Gram-negative strains and that of (E)-cinnamaldehyde was 0.25 mg/ml for* S. aureus*, with the essential oil of* C. cassia* having an MIC of 0.6 mg/ml against* S. aureus*.

In a study by [40], (E)-cinnamaldehyde was tested against ten Gram-positive and Gram-negative bacterial strains and three fungal species. The results demonstrated an inhibitory effect, according to the classification established by [41], with MIC values ranging from 0.78 to 12.5 *μ*l/ml for* S. aureus, E. coli,* and* P. aeruginosa*, bacteria which were used in the present study; the specific values for these concentrations were 1.56 and 12.5 *μ*l/ml, respectively. In the present study, the MIC values for (E)-cinnamaldehyde against* S. aureus, E. coli* and* P. aeruginosa* were 0.25 and 0.50 mg/ml, respectively, corresponding to approximately, 0.24 and 0.48 *μ*l/ml. Thus, the observed inhibitory effect obtained in this study was up to approximately twice as high as those observed by [40].

A study by [42] demonstrated the antibacterial activity of cinnamaldehyde against* E. coli* and* S. aureus*. In this study, cinnamaldehyde presented MIC values of 0.25 *μ*l/m and MBC value of 0.5 *μ*l/ml for both bacteria, similar to the data obtained in this study. In addition, scanning electron microscopy showed morphological changes, which were confirmed by the increase of nucleic acid and protein levels in the cell suspension, indicating that the cell membrane was damaged. Thus, according to the authors, cinnamaldehyde plays a role in disrupting the bacterial cell membrane.

### 3.3. Antibiofilm Activity of the Essential Oils from the Trunk Bark of* C. zeylanicum* and* C. cassia *and Cinnamaldehyde

#### 3.3.1. Quantification of Biofilm Biomass and Cell Viability in Biofilms

The biofilm biomass quantification data are shown in Table 3.

According to the results presented (see Table 3), the comparison between the concentrations of the substances that reduce* S. pyogenes* biofilms shows that the (E)-cinnamaldehyde concentration was approximately eight times higher than that of EOCc and four times higher than that of EOCz. In this case, it may be suggested that other components of the essential oils also participate synergistically in the antibiofilm action. A similar behavior is observed on biofilms of* P. aeruginosa*. However, against* Staphylococcus*, the concentration of the essential oils remains constant when compared to (E)-cinnamaldehyde or is twice as high as that of* S. epidermidis* biofilm.

**Figure 3:**
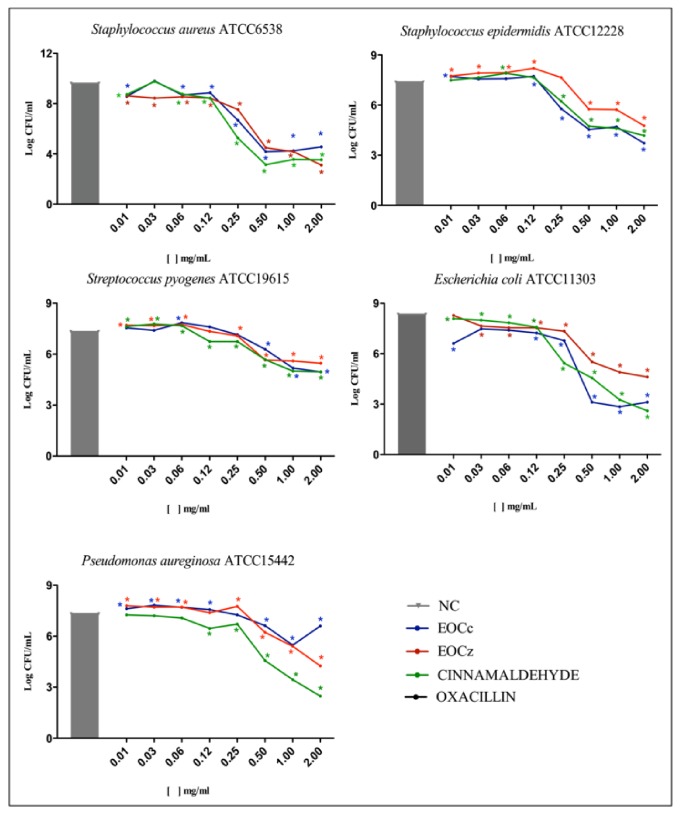
Cell viability in bacterial biofilms after the action of the essential oils from* C. cassia* and* C. zeylanicum *stems, cinnamaldehyde and oxacillin (mg/ml). *∗* p<0.01. NC: negative control; EOCc: essential oil* Cinnamomum cassia*; EOCz: essential oil* Cinnamomum zeylanicum*.

The cell viability verification data in bacterial biofilms are presented in Figure 3.

Commercially obtained* C. zeylanicum* essential oil was able to inhibit the biomass and number of viable* P. aeruginosa* biofilm cells at concentrations from 0.12 to 1.92 mg/ml. At 1.92 mg/ml, no biomass was observed and the viable cell reduction was significantly different from that of the negative control [43]. One study showed that 41.7% of biofilms of* P. aeruginosa* and 33.3% of* S. aureus* biofilms were sensitive to* C. zeylanicum* oil [44].

Another* Cinnamomum *species also exhibited antibiofilm activity; the essential oil of the trunk bark of* C. burmannii* was used to inhibit planktonic cell growth and the development of* S. aureus* and* P. aeruginosa* biofilms. These results showed that, at a concentration of 0.12% (v/v), the oil was able to inhibit planktonic cell growth of both bacteria by 50%. Fifty percent inhibition of biofilm formation was observed at a concentration of 0.03%, while at a concentration of 0.12% (v/v), biofilms formed by both bacteria were destabilized [45].

One study demonstrated that trans-cinnamaldehyde inhibited* E. coli* biofilm formation in urinary catheter fragments. Biofilm prevention was tested using catheter fragments inoculated with* E. coli* and treated with trans-cinnamaldehyde (0, 0.1, 0.25, and 0.5%) for 0, 1, 3, and 5 days. All of the concentrations assayed prevented* E. coli* biofilm formation in urinary catheter fragments [46].

## 4. Conclusions

Essential oils and (E)-cinnamaldehyde inhibit the growth of Gram-positive and Gram-negative bacteria in planktonic form. In addition, they inhibit the formation of biofilms, which are directly related to infections. The most susceptible biofilms were* P. aeruginosa* and* E. coli* microorganisms. Bioactivity may be associated with the presence of high content of (E)-cinnamaldehyde in the composition of essential oils. Therefore, these essential oils and their major component may be considered as possible sources for the development of new antimicrobial agents and may be used in synergy with currently available synthetic antibiotics or antimicrobials. In addition, the (E)-cinnamaldehyde molecule is promising as a prototype for derivatives with antibacterial properties and extended antibiotics.

## Figures and Tables

**Table 1:** Chemical composition, retention index experimental (RIExp), retention index of the literature (RILit), and percentage of the identified components (%) from the essential oils of *C. zeylanicum* (EOCz) and *C. cassia* (EOCc) stems.

**Compounds**	**R** **I**_**Exp**_	**R** **I**_**Lit**_	**EOCz**	**EOCc**
*α*-Thujene	920	930	0.08	-^a^
*α*-Pinene	926	939	0.48	-
Camphene	938	954	0.21	-
Benzaldehyde	948	960	0.20	1.86
*β*-Pinene	956	979	0.16	-
*α*-Phellandrene	973	1002	0.45	-
Z-*β*-Ocimene	979	1037	0.35	-
p-Cimene	984	1024	0.98	-
Limonene	986	1029	0.34	-
1,8-Cineole	988	1031	2.44	-
Salicylaldehyde	997	1044	-	0.53
E-*β*-Ocimene	1010	1050	0.08	-
Acetophenone	1035	1065	0.32	-
Terpinolene	1075	1088	0.07	-
Linalool	1104	1096	4.23	-
Phenyl ethyl alcohol	1121	1107	-	0.18
Coahuilensol	1171	1170	0.20	0.98
4-Terpineol	1187	1177	0.27	-
*α*-Terpineol	1200	1188	0.32	-
Z-Cinnamaldehyde	1228	1219	0.35	-
Hydrocinnamic alcohol	1239	1224	0.09	-
**E-Cinnamaldehyde**	1293	1270	**68**.**71**	**90**.**22**
Safrole	1298	1287	0.08	-
E-Cinnamyl alcohol	1316	1304	0.31	0.31
**Eugenol**	1363	1356	**6**.**33**	-
*α*-Copaene	1381	1376	1.82	0.32
E-Caryophyllene	1426	1419	1.85	-
Cumarin	1454	1434	-	1.37
**E-Cinnamoyl acetate**	1456	1446	**7**.**12**	2.04
*α*-Humulene	1463	1454	0.37	-
*γ*-Muurolene	1481	1479	-	0.15
Ar-Curcumene	1486	1480	-	0.07
*α*-Muurolene	1504	1500	-	0.10
*β*-Bisabolene	1512	1505	-	0.23
*δ*-Amorphene	1521	1512	0.09	0.12
Eugenyl acetate	1524	1522	0.32	-
E-Methoxi-cinnamaldehyde	1541	1528	-	0.73
E-Nerolidol	1566	1563	-	0.21
Caryophyllene oxide	1591	1583	0.24	0.11
Tetradecanal	1618	1612	-	0.09
Benzoyl benzoate	1779	1760	0.99	-

**Total**			99.85	99.62

^a^Not detected.

**Table 2:** Minimum inhibitory concentration (MIC) and minimum bactericidal concentration (MBC) values of essential oils from *C. zeylanicum* and *C. cassia* stems, (E)-cinnamaldehyde and oxacillin (mg/ml) on bacteria.

Microorganisms	**EOCz**		**EOCc**		**(E)-Cinnamaldehyde**		**Oxacillin**	
	MIC	MBC	MIC	MBC	MIC	MBC	MIC	MBC
*S. aureus*	0.50	0.50	0.25	1.00	0.25	0.50	-	-
*S. epidermidis*	0.50	1.00	0.25	1.00	0.25	1.00	-	-
*S. pyogenes*	0.50	-	0.50	-	0.50	-	-	-
*P. aeruginosa*	0.50	-	0.50	-	0.50	-	-	-
*E. coli*	0.50	2.00	0.25	-	0.25	2.00	-	-

**Table 3:** Minimum concentration of essencial oil from *C. cassia* (EOCc) and *C. zeylanicum* (EOCz) stems, (E)-cinnamaldehyde and oxacillin (mg/ml) reduce biofilm biomass by 100% in comparison to normal biofilm growth.

	**Substances**			
**Microorganisms **	**EOCc**	**EOCz**	**(E)-cinnamaldehyde**	**Oxacillin**
*S. aureus*	0.25	0.25	0.25	0.01
*S. epidermidis*	0.50	0.50	0.25	0.01
*S. pyogenes*	0.06	0.12	0.50	0.01
*P. aeruginosa*	0.06	0.12	0.25	0.01
*E. coli*	0.12	0.50	0.25	0.01

## Data Availability

The data used to support the findings of this study are available from the corresponding author upon request.

## References

[1] Hurlow J., Couch K., Laforet K., Bolton L., Metcalf D., Bowler P. (2015). Clinical biofilms: a challenging frontier in wound care. *Advances in Wound Care*.

[2] Cortes M., Consuegra J., Sinisterra R. (2011). *Biofilm formation, control and novel strategies for eradication,” Science against microbial pathogens: communicating current research and technological advances*.

[3] Vasudevan R. (2014). Biofilms: microbial cities of scientific significance. *Journal of Microbiology & Experimentation*.

[4] Hall-Stoodley L., Costerton J. W., Stoodley P. (2004). Bacterial biofilms: from the natural environment to infectious diseases. *Nature Reviews Microbiology*.

[5] Batoni G., Maisetta G., Brancatisano F. L., Esin S., Campa M. (2011). Use of antimicrobial peptides against microbial biofilms: Advantages and limits. *Current Medicinal Chemistry*.

[6] Zijnge V., van Leeuwen M. B., Degener J. E. (2010). Oral Biofilm Architecture on Natural Teeth. *PLoS ONE*.

[7] Bjarnsholt T., Jensen P. Ø., Fiandaca M. J. (2009). Pseudomonas aeruginosa biofilms in the respiratory tract of cystic fibrosis patients. *Pediatric Pulmonology*.

[8] Bjarnsholt T., Kirketerp-Møller K., Jensen P. Ø. (2008). Why chronic wounds will not heal: a novel hypothesis. *Wound Repair and Regeneration*.

[9] Homøe P., Bjarnsholt T., Wessman M., Sørensen H. C. F., Johansen H. K. (2009). Morphological evidence of biofilm formation in Greenlanders with chronic suppurative otitis media. *European Archives of Oto-Rhino-Laryngology*.

[10] Waar K., Degener J. E., van Luyn M. J., Harmsen H. J. M. (2005). Fluorescent *in situ* hybridization with specific DNA probes offers adequate detection of *Enterococcus faecalis* and *Enterococcus faecium* in clinical samples. *Journal of Medical Microbiology*.

[11] Li H., Wang D., Sun X., Hu L., Yu H., Wang J. (2012). Relationship between bacterial biofilm and clinical features of patients with chronic rhinosinusitis. *European Archives of Oto-Rhino-Laryngology*.

[12] Reid G., Potter P., Delaney G., Hsieh J., Nicosia S., Hayes K. (2000). Ofloxacin for the treatment of urinary tract infections and biofilms in spinal cord injury. *International Journal of Antimicrobial Agents*.

[13] Shi G.-S., Boost M., Cho P. (2015). Prevalence of antiseptic-resistance genes in staphylococci isolated from orthokeratology lens and spectacle wearers in Hong Kong. *Investigative Ophthalmology & Visual Science*.

[14] Macfarlane S., Dillon J. F. (2007). Microbial biofilms in the human gastrointestinal tract. *Journal of Applied Microbiology*.

[15] Onanuga A., Awhowho G. O. (2012). Antimicrobial resistance of Staphylococcus aureus strains from patients with urinary tract infections in Yenagoa, Nigeria. *Journal of Pharmacy and Bioallied Sciences*.

[16] Balamurugan K. (2015). HIF-1 at the crossroads of hypoxia, inflammation, and cancer. *International Journal of Cancer*.

[17] Gupta P., Sarkar S., Das B., Bhattacharjee S., Tribedi P. (2016). Biofilm, pathogenesis and prevention—a journey to break the wall: a review. *Archives of Microbiology*.

[18] Takaine M., Imada K., Numata O., Nakamura T., Nakano K. (2014). The meiosis-specific nuclear passenger protein is required for proper assembly of forespore membrane in fission yeast. *Journal of Cell Science*.

[19] Petrovska B. B. (2012). Historical review of medicinal plants' usage. *Pharmacognosy Reviews*.

[20] Bakkali F., Averbeck S., Averbeck D., Idaomar M. (2008). Biological effects of essential oils—a review. *Food and Chemical Toxicology*.

[21] Burt S. (2004). Essential oils: their antibacterial properties and potential applications in foods. *International Journal of Food Microbiology*.

[22] Kordali S., Kotan R., Mavi A., Cakir A., Ala A., Yildirim A. (2005). Determination of the chemical composition and antioxidant activity of the essential oil of *Artemisia dracunculus* and of the antifungal and antibacterial activities of Turkish *Artemisia absinthium*, *A. dracunculus*, *Artemisia santonicum*, and *Artemisia spicigera* essential oils. *Journal of Agricultural and Food Chemistry*.

[23] Borges A., Abreu A. C., Dias C., Saavedra M. J., Borges F., Simões M. (2016). New perspectives on the use of phytochemicals as an emergent strategy to control bacterial infections including biofilms. *Molecules*.

[24] Lima M. P., Zoghbi M. G., Andrade M. B. (2005). Volatile constituents from leaves and branches of zeylanicum (Lauraceae). *Acta Amazon*.

[25] Silva K. B., Alves E. U., Bruno R. d., Santos S. d., Barroso L. M. (2012). Tolerância à dessecação de sementes de Cinnamomum zeylanicum Ness. *Semina: Ciências Agrárias*.

[26] Hassan S. A., Barthwal R., Nair M. S. (2012). Aqueous bark extract of *Cinnamomum zeylanicum*: a potential therapeutic agent for streptozotocin- induced type 1 diabetes mellitus (T1DM) rats. *Tropical Journal of Pharmaceutical Research*.

[27] Sousa M. P., Matos M. E. O., Matos F. J. A., Machado M. I. L., Craveiro A. A., UFC (2004). *Constituintes químicos ativos e propriedades biológicas de Plantas Medicinais Brasileiras*.

[28] Luo Q., Wang S.-M., Lu Q., Luo J., Cheng Y.-X. (2013). Identification of compounds from the water soluble extract of cinnamomum cassia barks and their inhibitory effects against high-glucose-induced mesangial cells. *Molecules*.

[29] Chaudhry N. M. A., Tariq P. (2006). Anti-microbial activity of Cinnamomum cassia against diverse microbial flora with its nutritional and medicinal impacts. *Pakistan Journal of Botany*.

[30] Brasil Agência Nacional de Vigilância Sanitária (2010). *Farmacopeia brasileira*.

[31] Almeida D., Cavalcanti Y., Castro D., Lima O. (2012). Atividade Antifúngica e Alterações Morfológicas Induzidas pelo Óleo Essencial de Cinnamomum cassia frente Cepas de Candida albicans Isoladas de Pacientes HIV Positivos. *Pesqui Bras Odontopediatria Clin Integr*.

[32] Chen W., Golden D. A., Critzer F. J., Davidson P. M. (2015). Antimicrobial activity of cinnamaldehyde, carvacrol, and lauric arginate against salmonella Tennessee in a glycerol-sucrose model and peanut paste at different fat concentrations. *Journal of Food Protection*.

[33] van Den Dool H., Dec. Kratz P. (1963). A generalization of the retention index system including linear temperature programmed gas—liquid partition chromatography. *Journal of Chromatography A*.

[34] NCCLS (2003). *Methods for Dilution Antimicrobial Susceptibility Tests for Bacteria that Grow Aerobically*.

[35] Sá N. C., Cavalcante T. T. A., Araújo A. X. (2012). Antimicrobial and antibiofilm action of casbane diterpene from *Croton nepetaefolius* against oral bacteria. *Archives of Oral Biolog*.

[36] Majid A., Javad A., Hosseint (2015). The effect of zeylanicum oil on chemical characteristics of Lyoner- type sausage during refrigerated storage. *Veterinary Research Forum*.

[37] Zeng J.-F., Zhu H.-C., Lu J.-W., Hu L.-Z., Song J.-C., Zhang Y.-H. (2017). Two new geranylphenylacetate glycosides from the barks of Cinnamomum cassia. *Natural Product Research (Formerly Natural Product Letters)*.

[38] Anwar F., Hussain A. I., Sherazi S. T. H., Bhanger M. I. (2009). Changes in composition and antioxidant and antimicrobial activities of essential Oil of fennel (foeniculum vulgare mill.) fruit at different stages of maturity. *Journal of Herbs, Spices & Medicinal Plants*.

[39] Ooi L. S. M., Li Y., Kam S.-L., Wang H., Wong E. Y. L., Ooi V. E. C. (2006). Antimicrobial activities of Cinnamon oil and cinnamaldehyde from the Chinese medicinal herb *Cinnamomum cassia* Blume. *American Journal of Chinese Medicine*.

[40] Sanla-Ead N., Jangchud A., Chonhenchob V., Suppakul P. (2012). Antimicrobial activity of cinnamaldehyde and eugenol and their activity after incorporation into cellulose-based packaging films. *Packaging Technology and Science*.

[41] Elgayyar M., Draughon F. A., Golden D. A., Mount J. R. (2001). Antimicrobial activity of essential oils from plants against selected pathogenic and saprophytic microorganisms. *Journal of Food Protection*.

[42] Zhang L.-Q., Zhang Z.-G., Fu Y., Xu Y. (2015). Research progress of trans-cinnamaldehyde pharmacological effects. *China Journal of Chinese Materia Medica*.

[43] Coelho F. A. B. L., Pereira M. O., Mendez-Vilas A. (2013). Exploring new treatment strategies for Pseudomonas aeruginosa biofilm infections based on plant essential oils. *Microbial Pathogens and Strategies for Combating Them: Science, Technology and Education*.

[44] Sambyal S. S., Sharma P., Shrivastava D. (2017). Anti-biofilm Activity of Selected Plant Essential Oils against Pseudomonas aeruginosa and Staphylococcus aureus. *International Journal of Current Microbiology and Applied Sciences*.

[45] Pratiwi S. U. T., Lagendijk E. L., de Weert S., Idroes R., Hertiani T., Van den Hondel C. (2015). Effect of Cinnamomum burmannii Nees ex Bl. and Massoia aromatica Becc. Essential oils on planktonic growth and biofilm formation of Pseudomonas aeruginosa and Staphylococcus aureus In Vitro. *International Journal of Applied Research in Natural Products*.

[46] Kot B., Wicha J., Piechota M., Wolska K., Grużewska A. (2015). Antibiofilm activity of trans-cinnamaldehyde, p-coumaric, and ferulic acids on uropathogenic Escherichia coli. *Turkish Journal of Medical Sciences*.

